# Reduced serum AHR agonistic activity reflects amyloid dysregulation in AT1 subtypes of Alzheimer’s disease

**DOI:** 10.1186/s13195-026-01978-w

**Published:** 2026-02-06

**Authors:** Thanos Tsaktanis, Laura Rudtke, Leander Ammon, Stefan Mestermann, Veit Rothhammer, Piotr Lewczuk, Juan Manuel Maler, Johannes Kornhuber, Timo Jan Oberstein

**Affiliations:** 1 Department of Neurology, Universitätsklinikum Erlangen, Friedrich-Alexander-Universität Erlangen-Nürnberg (FAU), Erlangen, 91054 Germany; 2 Department of Psychiatry and Psychotherapy, Universitätsklinikum Erlangen, Friedrich-Alexander-Universität Erlangen-Nürnberg (FAU), Schwabachanlage 6, Erlangen, 91054 Germany; 3 Department of Child and Adolescent Mental Health, Universitätsklinikum Erlangen, Friedrich‐Alexander‐Universität Erlangen‐Nürnberg (FAU), Erlangen, Germany; 4 Deutsches Zentrum Immuntherapie (DZI), Universitätsklinikum Erlangen, Erlangen, 91054 Germany; 5 Department of Neurodegeneration Diagnostics, Medical University of Bialystok, and Department of Biochemical Diagnostics, University Hospital of Bialystok, Białystok, 15-267 Poland

**Keywords:** Alzheimer's disease (AD), Inflammation, Aryl hydrocarbon receptor (AHR), Amyloid beta metabolism, ATN classification

## Abstract

**Background:**

The aryl hydrocarbon receptor (AHR) is a ligand-activated transcription factor linking environmental signals to immune regulation and neurodegeneration. While AHR agonists exert protective effects in Alzheimer’s disease (AD) models, their relevance in humans remains unclear. We investigated AHR agonistic activity in serum and cerebrospinal fluid (CSF) across AT1-defined AD subtypes and its association with amyloid beta (Aβ) metabolism.

**Methods:**

We measured AHR agonistic activity using a luciferase reporter assay in serum and cerebrospinal fluid (CSF) from 138 individuals (113 non-demented, 25 demented) classified by CSF Aβ1-42, Aβ1-42/Aβ1-40 ratio and pTau181 into four AT1 groups: A + T1 + (AD), A + T1- (isolated decrease of Aβ1-42/Aβ1-40 or Aβ1-42), A-T1 + (isolated tau pathology/PASSED), and A-T1- (biomarker-negative controls).

**Results:**

Serum AHR agonistic activity was significantly reduced in biomarker-positive individuals compared to controls (Δ z-score = 2.25, 95% CI [1.33, 3.17], *p* < 0.001), with the most pronounced reductions observed in two distinct phenotypes: non-demented A-T1 + individuals (z-score = -2.33, 95% CI [-3.26, -1.40]) and A + T1 + patients with preserved CSF Aβ1-42 despite pathological Aβ1-42/Aβ1-40 ratios (*p =* 0.006 compared to A + T1 + with pathological Aβ1-42). Serum AHR activity correlated negatively with CSF Aβ1-40 (ρ = -0.31, *p* < 0.001) and Aβ1-42 (ρ = -0.20, *p =* 0.021) across all participants. The relationship between pTau181 and serum AHR activity showed significant effect modification by amyloidopathy (A) (interaction *p* < 0.0001): negative in A- individuals (β = -0.54, *p* < 0.0001) but positive at high pTau181 levels in A + cases (β = 0.12, *p =* 0.025). CSF AHR activity showed no significant differences between AT1 groups.

**Conclusions:**

Peripheral AHR agonistic activity is reduced in early and atypical AD phenotypes and correlates with amyloid peptides Aβ1-42 and Aβ1-40 in CSF. The amyloid status-dependent reversal of pTau181-AHR associations suggests distinct regulatory mechanisms across AT1 groups.

**Supplementary Information:**

The online version contains supplementary material available at 10.1186/s13195-026-01978-w.

## Background

Genetic and environmental influences are crucial in shaping the immune response, affecting both overall health and the development of diseases, including neurodegenerative conditions such as Alzheimer’s disease (AD) [1, 2]. A possible link between environmental factors and the onset of Alzheimer's disease could be the aryl hydrocarbon receptor (AHR), a transcription factor activated by specific ligands. It plays a pivotal role in regulating aspects of both innate and adaptive immune responses within the peripheral immune system and the central nervous system (CNS), which has been demonstrated for multiple sclerosis in particular [3–7]. AHR agonists, which are biologically active molecules binding to this receptor, can be derived from environmental pollutants, dietary intake, the body’s own metabolic processes, and interactions with gut microbiota, making them a possible target for explaining the beneficial effects of the Mediterranean diet on Alzheimer's disease [2, 8, 9]. Recent evidence from an AD mouse model supports a protective role of the AHR agonistic activity via the induction of Amyloid beta (Aβ) degrading enzyme neprilysin [10]. Despite these promising aspects, we have not found any literature exploring the potential relationship between AHR activity and Alzheimer’s disease (AD) in human studies. This is particularly significant because most current AD research relies heavily on artificial mouse models, making human data crucial for a proper interpretation of AD pathology.

The diagnosis of AD is based on the detection of surrogate markers for the core neuropathologic changes in AD, amyloid beta (Aβ) plaques and neurofibrillary tangles, such as decreased Aβ1–42 and decreased ratio of Aβ1–42/Aβ1–40 (A) or increased phospho-tau (T) in the cerebrospinal fluid (CSF) [11]. The diagnoses of AD over two properties imply that besides the unambiguous cases, in this case A-T1- and A + T1 +, there are also cases with incongruent altered biomarkers, such as A + T1- and A-T1 +. In particular, the A-T1 + variant is found in about 20% of patients with mild amnesic syndrome in the sense of subjective cognitive decline (SCI) or mild cognitive decline (MCI) in memory clinics and has a more favorable prognosis and less longitudinal brain atrophy than the A + T1 + constellation [12]. This has led to the proposal to consider the A-T1 + constellation as a suspected non-Alzheimer´s disease pathophysiology (SNAP) [13]. Conversely, A-T1 + patients with elevated phosphotau181 in the CSF do not demonstrate a decline in Aβ1–42, as observed in AD, but rather, an increase in both Aβ1–42 and Aβ1–40 [12]. Thus, even if the A-T1 + constellation was independent of Alzheimer's disease, it remain associated with markers of amyloidopathy, which led us to describe individuals with the A-T1 + constellation more precisely as subjects with increased pTau-levels and Aβ surge with subtle cognitive deterioration (PASSED) [12, 14]. Changes in Aβ levels are known not only in neurodegenerative diseases; for example, in meningitis or multiple sclerosis (MS), there is a uniform reduction in Aβ1–42 and 1–40 in the cerebrospinal fluid [15, 16]. This also suggests an interplay between inflammation and AD core marker pathology, especially since immunological changes appear to play a role in early stages of AD [11]. In this study, we aimed to investigate the role of AHR in AD by assessing serum and CSF AHR agonistic activity across different stages of the disease in a cohort of cross-sectional patient samples. Our focus was on patients with varying levels of cognitive impairment and the relationship between AHR agonistic activity and the core biomarkers for AD.

## Materials and methods

### AHR ligand measurement

HEK293 cells were used in the transient transfection system, as previously described [17, 18]. In brief, 20,000 cells per well were plated in 96 well flat bottom plates. 24 h after plating, cells were transfected with equal amounts of pGud-Luc [19] and pTK-Renilla (Renilla luciferase under control of constitutively active Thymidine kinase promoter, Promega) using Fugene-HD Transfection Reagent (Promega) as suggested by the manufacturer. After 24 h, transfected cells were incubated with DMEM supplemented with 10% of patient serum in duplicates. Luciferase activity was analyzed 24 h later using the Dual Luciferase Reporter System (Promega). Firefly luciferase activity was normalized to Luciferase activity to determine relative AHR agonistic activity. The experiments were carried out in three batches and repeated three times each. The first group (*n* = 73) was used as the standard database and the values of AHR agonistic activity in all datasets were normalised to z-scores using the mean (0.2571) and standard deviation (0.03029).

### Data and sample acquisition

This retrospective cohort analysis was conducted on patients admitted to the Department of Psychiatry at the University Hospital Erlangen between May 2021 until January 2024. The inclusion criteria required patients to meet the following conditions: (i) a history of subjective or observed cognitive decline, (ii) a comprehensive assessment of cognitive function using the German version of the Consortium to Establish a Registry for Alzheimer's Disease neuropsychological battery plus (CERAD-NB +) [20], and (iii) the availability of a brain scan, preferably a magnetic resonance imaging (MRI) scan, performed within six months before or after hospital admission.

During routine diagnostic procedures, cerebrospinal fluid (CSF) and serum samples were collected and analyzed using validated dementia biomarkers. Additional samples were preserved at −80 °C following a standardized protocol to ensure their integrity for future analyses.

Baseline characteristics – including demographic data, medical history, comorbidities, neuropsychological profiles, years of education and clinical symptoms – were systematically collected at the time of study enrollment.

CSF neurodegenerative disease (NDD) biomarker levels, including Aβ1–40, Aβ1–42, the Aβ1–42/Aβ1–40 ratio, pTau181, and t-tau, were retrieved from a prospective laboratory database. The biomarkers were analyzed in the Laboratory for Clinical Neurochemistry and Neurochemical Dementia Diagnostics at the Department of Psychiatry using the Lumipulse G1200 platform from Fujirebio Europe (catalog # 230350, 230336, 231524, 230312, Gent, Belgium) in accordance with the protocols from the assay vendors. The transition of laboratory protocols, including changes in reference ranges, was carefully monitored and validated according to ISO 15189 standards.

Individuals in the control group had only subjective or mild cognitive impairment and had normal dementia markers, i.e. normal Aβ1–42 (> 570 pg/ml), Aβ1–40/Aβ1–42 ratio (> 0.06 pg/ml), pTau181 (< 50 pg/ml) and t-tau (< 415 pg/ml). For the other groups, i.e. A + T1 +, A-T1 +, A + T1-, the total tau level was not used for grouping. Consistent with the 2024 NIA-AA framework, we use T1 to denote pTau markers, in this case CSF pTau181, that are supposed to reflect soluble tau fragments related to Aβ plaque biology; ‘T2’ (tau PET/tau aggregates) was not assessed in this study.

Glial fibrillary acidic protein (GFAP) in human plasma was quantified by chemiluminescent immunoassay on the semi-automated LUMIPULSE G1200 analyzer (catalog # 261255, Fujirebio Europe, Ghent, Belgium) according to the manufacturer’s instructions. Within-run precision was assessed using a pooled plasma sample from eight individuals. The intra-assay coefficient of variation (CV) was below 2.5% across 10 replicate measurements.

Routine laboratory parameters reflecting renal, hepatic and metabolic function (including creatinine, estimated glomerular filtration rate [eGFR], GPT, GOT, γ-GT, and HbA1c) were obtained from standardized clinical chemistry analyses performed as part of routine diagnostic evaluation at the University Hospital Erlangen.

In human serum, C-reactive protein (CRP) was measured by immunoturbidimetric assay, while IL-6 and TNF-α were quantified by standardized immunoassays in the central clinical laboratory of the University Hospital Erlangen following established quality management procedures.

### Statistics

To facilitate comparability of effect sizes, continuous variables were z-standardised within the analytic sample. Skewed markers (e.g. IL-6) were analysed on the log scale prior to z-scoring. The normal distribution of the data was assessed using the Shapiro–Wilk test, and the homogeneity of variance was tested with Levene's test. The psychometric test statistics employed in this study were using with age-, sex-, and education-adjusted z-scores. Differences between the selected AT1 groups were analyzed using Pearson's χ^2^ test for categorical variables (such as sex), or the Kruskal–Wallis H-test followed by Dunn's multiple comparison test for ordinal or non-normally distributed data (e.g., MMSE). Where appropriate, p-values obtained using permutation tests (5,000 label shuffles) were employed to stabilise inference under small n and mild non-normality. Estimates are reported alongside bias-corrected and accelerated (BCa) 95% bootstrap confidence intervals (based on 5,000 resamples). For continuous variables, the t-test, analysis of variance (ANOVA), analysis of covariance (ANCOVA) or the Brown-Forsythe test for inhomogeneous variances was applied, followed by Tukey B or Dunn´s multiple comparison test with Bonferroni correction if a significant effect was found (e.g., age, education, pTau-, tTau-, Aβ42-, Aβ40-levels (pg/ml), Aβ1–42/Aβ1–40 ratio, AHR agonistic activity). As additional sensitivity analyses, we repeated broad A ±/T1 ± comparisons excluding values within ± 0.10 of the clinical cut-offs for Aβ1–42/Aβ1–40 and pTau181. Linear and rank associations between AHR agonistic activities (in serum and CSF) and candidate markers (Aβ1–40, Aβ1–42, the Aβ1–42/Aβ1–40 ratio, pTau181, GFAP, CRP and IL-6 and TNF-α) were summarised using Pearson's r and Spearman's ρ, with missing data being handled via pairwise deletion. To ensure robustness, 95% confidence intervals (CIs) were obtained via bootstrap resampling (5,000 resamples). To test whether amyloid pathology modified biomarker-AHR associations, we performed a two-step analytical approach: (1) formal interaction testing using linear regression models with biomarker × Aβ1–42/Aβ1–40 ratio status (normal vs. pathological) interaction terms, and (2) stratified analyses when significant interactions were detected. Linear regression models included the same covariates as GAM models (described below) but with parametric age effects. Significant interactions (*p* < 0.05) indicated amyloid-dependent effect modification warranting separate analysis by amyloid status (A + vs A-). To allow for potential non-linear relations between CSF surrogate markers and serum AHR agonistic activity, generalized additive models (GAMs) were fitted (mgcv package in R) with thin-plate regression splines. Age (smooth), sex, education, eGFR and GPT (representatives of renal/hepatic function), HbA1c, and MMSE were prespecified covariates. For biomarkers showing significant amyloid-dependent effects, separate GAMs were fitted for A- and A + groups to characterize potentially different functional forms. For biomarkers without significant interactions (i.e., Aβ1–40, Aβ1–42), pooled GAMs with amyloid status as a covariate were used.

Unless stated otherwise, renal and hepatic adjustment used eGFR and GPT as representatives (sensitivity models used first principal component (PC1) scores from small principal component analyses of eGFR/creatinine and GPT/GOT/γ-GT). Smoothers were limited to k = 4 to avoid overfitting, estimated by REML (allowing data-driven penalization to zero) and a mild γ penalty = 1.4. Model fit is summarized by adjusted R^2^, deviance explained, AIC, and the effective degrees of freedom (edf), F-statistic, and p-value of each smooth. We inspected concurvity and parametric collinearity to ensure no instability from correlated covariates. Partial-effect curves (± 95% SE ribbons) were produced for visualization. For a sub-cohort of 86 individuals, we explicitly tested whether AHR activity reflects systemic or neuroinflammation. First, we examined whether astrocyte reactivity, as assessed by plasma GFAP levels, modulates the association between biomarkers and AHR agonistic activity. Participants were stratified into GFAP + and GFAP- groups using two approaches: median split and Youden index-derived cutoff. Linear regression models tested whether CSF biomarker × GFAP interactions predicted AHR agonistic activity (measured in both serum and CSF), with adjustment for age, education, sex, and MMSE. The robustness of findings was validated using continuous GFAP values and alternative percentile-based cutoffs (25th, 33rd, 67th, 75th percentiles). Additionally, we modeled serum AHR activity with GAMs two concurrent smooths,

$$\mathrm{AHRserum}(\mathrm{z})\sim \mathrm{s}(\text{Systemic PC}1)+\mathrm{s}(\mathrm{GFAP})+\mathrm{covariates}$$

where Systemic PC1 was the first principal component of CRP, IL-6, and TNF-α (z-scored), and covariates were age (smooth), sex, education, eGFR, GPT, HbA1c, and MMSE. Smoothers used thin-plate regression splines (k = 4), fitted by REML with select = TRUE (γ = 1.4). These analyses were planned to quantify evidence against an inflammation-driven AHR signal. We repeated the analysis with (i) CRP only (single smooth), (ii) GFAP only, and (iii) Systemic PC1 only; we also mirrored all inflammation models using AHR in CSF as the outcome, and re-specified renal/hepatic adjustment using PC1 axes instead of representative markers. Conclusions were considered consistent if smooth terms were penalized to ~ 0 edf and/or non-significant with unchanged AIC. Findings were considered consistent when point estimates and directions were stable across these re-specifications. Data analysis was performed using SPSS statistical software (version 28.0; SPSS, Chicago, IL, USA) and R (RStudio: Integrated Development Environment for R 2024.12.1+563 “Kousa Dogwood”, Boston, MA, USA).

## Results

### Study population

A total of 113 non-demented subjects and 25 demented subjects were included in this study. Among the non-demented subjects, 44 were A + T1 +, 9 were A + T1-, 25 were A-T1 +, and 35 were biomarker-negative controls (A-T1-). The four groups did not differ significantly in sex distribution, years of education or MMSE scores, but showed a significant age difference (*p =* 0.005), with the A + T1- group being the oldest and the A-T1 + group being the youngest (Table 1). However no pairwise age comparisons remained significant after Bonferroni correction for multiple testing at the *p* < 0.05 threshold. A table showing the exact distribution into three diagnostic categories: subjective cognitive impairment (SCI), mild cognitive impairment (MCI) and dementia and baseline characteristics of these AT1 groups is given in (Supplementary Table 1). The values of the Aβ42, Aβ40, Aβ1–42/Aβ1–40 ratio, pTau181 and total tau levels in the various non-demented AT1 subgroups are presented in (Table 1).

**Table 1:** Characteristics of individuals in non-demented AT1 subgroups

	**A-T1-**				**A + T1 + **				**A-T1 + **				**A + T1-**				***p*-value**
	**n**		**%**		**n**		**%**		**n**		**%**		**n**		**%**		
**n [female]**	35	17	31	15	44	21	39	19	25	12	22	11	9	5	8	4	0.979
	**mean**		**SD**		**mean**		**SD**		**mean**		**SD**		**mean**		**SD**		
Age at Visit [years]	65		8		70		10		65		8		74		9		0.005
Education [years]	14		3		14		4		13		3		14		5		0.791
MMSE	27		2		27		2		28		2		26		2		0.179
Aβ 1–42/Aβ 1–40	0.097		0.015		0.041		0.010		0.082		0.016		0.070		0.034		< 0.001
Aβ 1–42 [pg/ml]	1113		342		643		197		1442		372		449		166		< 0.001
Aβ 1–40 [pg/ml]	11,366		3104		16,775		6084		17,301		3432		8099		3512		< 0.001
pTau181 [pg/ml]	36		9		108		49		68		13		34		14		< 0.001
total tau [pg/ml]	250		77		719		450		447		336		224		76		< 0.001
GFAP [pg/ml]^a^	42		22		50		18		55		39		89		41		< 0.001

^a^n(GFAP) = 86, 34 A-T1-, 6 A + T1-, 11 A-T1 +, 35 A + T1 +

In addition to the differences between the surrogate parameters resulting from the grouping into the different AT1 groups alone, the non-demented A-T1 + group, in accordance with the PASSED definition, exhibited significantly elevated Aβ40 levels compared to biomarker-negative controls (M_Diff_ = 5935 pg/mL, 95% CI [4234, 7636], *p* < 0.001) and elevated Aβ42 levels (M_Diff_ = 330 pg/mL, 95% CI [143, 516], *p* < 0.001). By definition, the Alzheimer's disease group (A + T1 +) was characterized by pathologically elevated pTau181 and a decreased Aβ1–42 or Aβ1–42/Aβ1–40 ratio. As a result, the A + T1 + group consisted of two groups, one of 32 individuals with normal Aβ42 levels and pathological Aβ1–42/Aβ1–40 ratio, and one of 31 individuals with both pathological Aβ42 levels and Aβ1–42/Aβ1–40 ratio. The A + T1 + group with normal Aβ42 levels had higher Aβ40 levels than the A + T1 + group with pathological Aβ42 and the non-demented A-T1- controls, *p* < 0.01, M_Diff_ = 6843, 95% CI (4362, 9324) and *p* < 0.001 M_Diff_ = 7646, 95% CI (5460, 9832) (Supplementary Table 2).

### Serum AHR agonistic activity is reduced in CSF biomarker-positive cases and lowest in A − T1 + (PASSED)

**Fig. 1:**
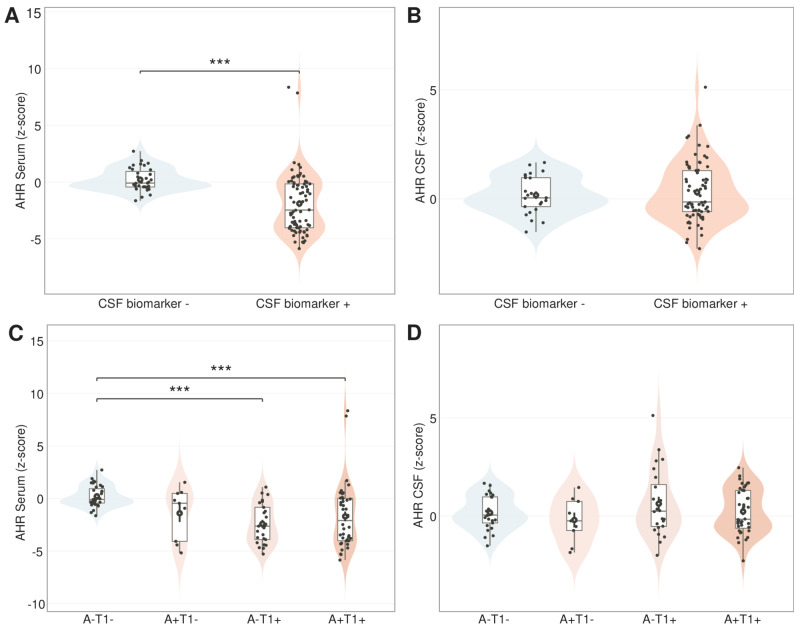
Serum and CSF AHR agonistic activity across CSF biomarker status and AT1 subgroups in non-demented individuals (**A-B**) Violin/box plots showing z-scored AHR agonistic activity in (**A**) serum and (**B**) CSF, comparing CSF biomarker-negative controls (*n* = 35) to biomarker-positive non-demented cases (*n* = 78; SCI/MCI). Jittered points represent individual participants; white dots indicate group means; box plots display median and interquartile range. Serum AHR agonistic activity was significantly reduced in biomarker-positive individuals compared to controls in covariate-adjusted analyses controlling for age, education, sex, and MMSE (Δ z-score = −2.25, 95% CI [−3.17, −1.33], *p* < 0.001. CSF AHR agonistic activity showed no significant difference between groups after covariate adjustment (Δ z-score = −0.15, 95% CI [−0.75, 0.45], *p =* 0.62). ***p < 0.001. **C-D** Violin/box plots showing z-scored AHR agonistic activity in (**C**) serum and (**D**) CSF across AT1 subgroups in non-demented individuals (A − T1 − : *n* = 35; A + T1 − : *n* = 9; A − T1 + : *n* = 25; A + T1 + : *n* = 44). One-way ANCOVA revealed a significant main effect of AT1 group on serum AHR activity after adjustment for age, education, sex, and MMSE (*p* < 0.001). Pairwise comparisons showed that both A − T1 + and A + T1 + groups had significantly lower serum AHR activity compared to A − T1 − controls (*p* < 0.001 and *p =* 0.001, respectively). CSF AHR agonistic activity showed no significant differences across AT1 groups. All analyses restricted to non-demented individuals. Detailed ANCOVA results and estimated marginal means are provided in Supplementary Table 3. ***p < 0.001; **p < 0.01; ns = not significant

In initial comparison of CSF biomarker-negative controls (*n* = 35) and biomarker-positive, non-demented cases (*n* = 78), serum AHR activity was significantly reduced in the biomarker-positive group (Difference in z-scores (Δ z-score) = −2.10, 95% CI [−3.01, −1.20], *p* < 0.001), which remained significant in a multiple regression analysis correcting for age, years of education, gender and MMSE (Δ z-score = −2.25, 95% CI [−3.17, −1.33], *p* < 0.001; Fig. 1A). A sensitivity analysis excluding values within ± 10% of the assay cut-off reduced the sample from 113 to 87 cases (26 excluded). Results remained significant in both unadjusted (Δ z-score = −1.77, 95% CI [−2.81, −0.72,], *p =* 0.001) and covariate-adjusted analyses (Δ z-score = −1.87, 95% CI [−2.99, −0.75], *p =* 0.001; adjusted for age, education, gender, and MMSE). In contrast, CSF AHR activity did not differ significantly between biomarker-negative and biomarker-positive groups after adjusting for age, education, sex, and cognitive status (Δ z-score = −0.15, 95% CI [−0.75, 0.45], *p =* 0.62; Fig. 1B). When stratifying by AT1 subgroup, a main effect of group was observed for serum AHR activity in non-demented individuals. One-way ANCOVA without covariates was significant (*p* < 0.001), and the effect remained after adjustment for age, years of education, sex, and MMSE (*p* < 0.001). Within the non-demented cohort the serum AHR agonistic activity was lowest in the A-T1 + group, with a z-score of −2.33, 95% CI [−3.26, −1.40], followed by the A + T1 + group, with a z-score of −1.81, 95% CI [−2.50, −1.11], the A + T1- group, with a z-score of −1.52, 95% CI [−3.05, 0.01], and the A-T1-N- group, with a z-score of 0.28, 95% CI [−0.49, 1.05] (Fig. 1C). In a pairwise comparison of the adjusted estimated marginal means of the one-way ANCOVA, the A-T1 + group and the A + T1 + group had lower serum AHR activity levels in non-demented subjects compared to the biomarker-negative control group, *p* < 0.001 and *p =* 0.001. AHR agonist activity in CSF showed no significant differences between AT1 groups, neither without covariates (*p =* 0.33) nor after adjustment for age, years of education, sex, and MMSE (*p =* 0.34; Fig. 1D). The results of the ANCOVAs, unadjusted and adjusted estimated marginal means are detailed in Supplementary Table 3.

### Serum AHR agonistic activity correlates negatively with CSF Aβ1–40 and CSF Aβ1–42 and with pTau181 depending on amyloid pathology

**Fig. 2:**
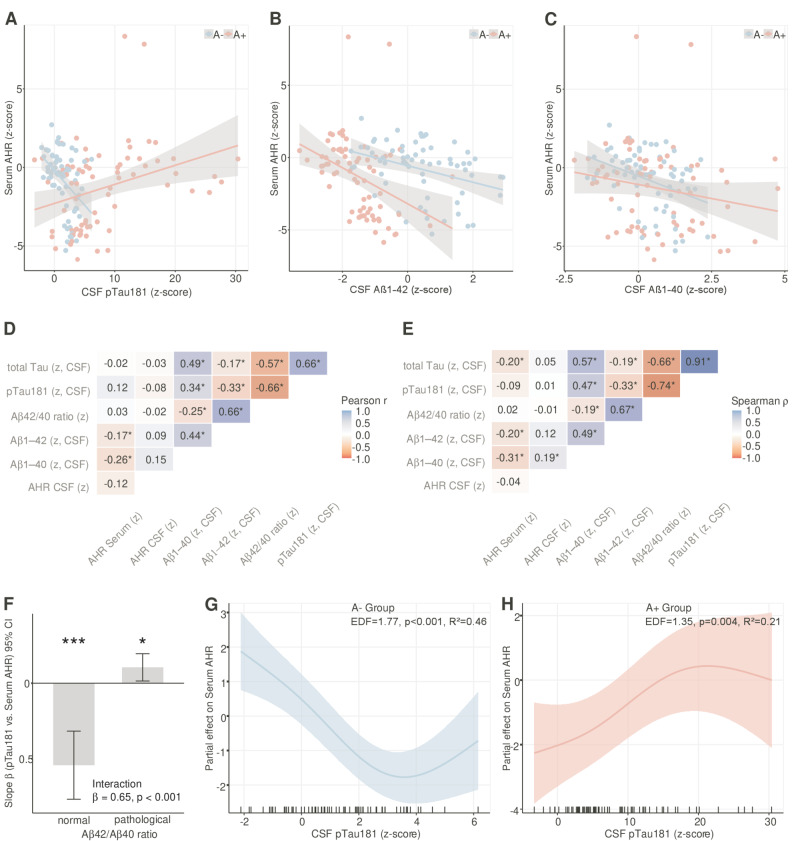
Correlation structure among serum and CSF AHR agonistic activity and CSF amyloid and tau markers. **A-C** Scatter plots illustrating the relationship between serum AHR z-score values and (**A**) CSF pTau181 (pg/ml), (**B**) CSF Aβ1–42 (pg/ml), and (**C**) CSF Aβ1–40 (pg/ml) in individuals with normal Aβ1–42/Aβ1–40 ratio (A-, blue) and pathological Aβ1–42/Aβ1–40 ratio (A +, red). Each point represents an individual subject. Solid lines represent smoothed conditional means with 95% confidence ribbons. **D-E** Upper-triangle heatmaps showing (**D**) Pearson's r and (**E**) Spearman's ρ among z-scored AHR serum, AHR CSF, Aβ1–40, Aβ1–42, Aβ1–42/Aβ1–40 ratio, pTau181, and total tau. Cells display correlation coefficients; asterisks denote *p* < 0.05 (two-sided; pairwise complete observations). These matrices illustrate inverse associations between Aβ1–40 or Aβ1–42 and serum AHR across the cohort, while the relationship between serum AHR and pTau181 is obscured in the overall sample due to opposing directional associations in A- and A + groups. **F** Interaction plot showing amyloid-dependent effect modification of the pTau181–serum AHR relationship. Linear regression revealed significant interaction between pTau181 and amyloid status (interaction β = 0.65, *p* < 0.001), with opposite directional effects: A- participants showed negative association (β = −0.54, SE = 0.12, *p* < 0.001, *R*^2^ = 0.46), while A + participants showed positive association (β = 0.11, SE = 0.05, *p =* 0.025, R^2^ = 0.21). Error bars represent 95% CI; ***p < 0.001, *p < 0.05. **G**- Partial effects of CSF pTau181 on serum AHR from separate GAMs for (**G**) A- group and (**H**) A + group. In A- participants, GAM revealed a robust moderately non-linear inverse relationship across the entire measurable pTau range (z-score −2 to 6; EDF = 1.77, *p* < 0.001, adjusted *R*^2^ = 0.46, explaining 53.2% deviance). In A + participants, the relationship was more linear (EDF = 1.35, *p =* 0.004, adjusted *R*^2^ = 0.21, explaining 29.0% deviance), with the positive pTau–AHR association emerging predominantly at elevated pTau levels (z-score > 6). Ribbons show 95% pointwise confidence intervals. Rug plots along the x-axis indicate individual data points. All GAMs adjusted for age (smooth term), sex, education, eGFR, GPT, HbA1c, and MMSE

To investigate associations between AHR agonistic activity and CSF biomarkers, we performed correlation analyses followed by covariate-adjusted linear regression models and generalized additive models (GAMs). All models adjusted for age, sex, years of education, kidney function (creatinine), liver function (GPT), HbA1c, and MMSE (Supplementary Fig. 1). In a preliminary correlation analyses by amyloid status, participants without amyloid pathology (A-, *n* = 66), serum AHR activity showed consistent negative correlations with all measured CSF biomarkers: pTau181 (Spearman ρ = −0.45, *p* < 0.001; Fig. 2A), total tau (ρ = −0.47, *p* < 0.001), Aβ1–42 (ρ = −0.40, *p =* 0.001; Fig. 2B), and Aβ1–40 (ρ = −0.37, *p =* 0.002; Fig. 2C). In contrast, participants with amyloid pathology (A +, *n* = 72) showed a positive correlation between pTau181 and serum AHR (ρ = 0.35, *p =* 0.003), while maintaining negative correlations with Aβ1–42 (ρ = −0.49, *p* < 0.001) and Aβ1–42/Aβ1–40 ratio (ρ = −0.33, *p =* 0.005). When all participants were analyzed together, negative correlations with Aβ1–40 (Spearman ρ = −0.31, *p* < 0.001; Pearson *r* = −0.26, *p =* 0.002), Aβ1–42 (Spearman ρ = −0.20, *p =* 0.021; Pearson *r* = −0.17, *p =* 0.043) remained statistically significant, while the pTau relationship was obscured by the opposing group-specific effects. To test whether amyloid pathology modified biomarker-AHR associations, we fitted linear regression models with interaction terms for pTau181, Aβ1–42, and Aβ1–40 (Supplementary Table 4). For pTau181, we observed highly significant effect modification by amyloid status (interaction β = 0.65, *p* < 0.0001). In the A- group, pTau showed a negative association with serum AHR (β = −0.54, SE = 0.12, *p* < 0.0001), with the model explaining 46% of outcome variance (Fig. 2D). In contrast, A + participants showed a positive association (β = 0.11, SE = 0.05, *p =* 0.025). These opposite associations suggest distinct regulatory mechanisms between pTau181 and AHR agonist activity depending on amyloidopathy status. In contrast, both Aβ1–42 and Aβ1–40 showed a shared negative association with serum AHR across both amyloid groups (for Aβ1–42: A- β = −0.55, SE = 0.19, *p =* 0.006; A + β = −1.24, SE = 0.37, *p =* 0.002; interaction β = 0.69, *p =* 0.10; for Aβ1–40: A- β = −0.66, SE = 0.24, *p =* 0.008; A + β = −0.34, SE = 0.22, *p =* 0.128; interaction β = −0.32, *p =* 0.32). While the A + group showed a numerically stronger Aβ1–42 effect, the interaction term did not reach statistical significance (*p =* 0.10), and both groups showed the same negative direction. Further stratified GAM analyses revealed that in A- participants, the relationship between CSF-pTau181 and AHR agonist activity was robust across the entire measurable pTau range (z-score = −2 to 6; EDF = 1.77, *p* < 0.0001, Fig. 2E, Supplementary Table 5). In A + participants, however, the relationship was more complex: within the overlapping pTau range (z-score < 6), the smooth term was effectively linear (EDF = 0.00, *p =* 0.87), indicating no association at low-to-moderate pTau levels. The positive pTau-AHR relationship in A + emerged only at high pTau levels (z-score > 6, Fig. 2E, Supplementary Table 5).

### AHR agonistic activity is decreased in A + T1 + individuals with normal Aβ1–42

**Fig. 3:**
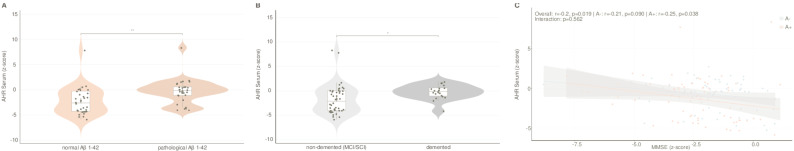
Serum AHR agonistic activity in relation to Aβ1–42 status, dementia status, and cognitive performance (**A**) Violin/box plot of serum AHR (z-score) in the A + T1 + subgroup, stratified by Aβ1–42 status (normal vs pathological per assay cut-off). Jittered points represent individual participants; white dots indicate group means; box plots show median and interquartile range. A + T1 + individuals (*n* = 32) with normal Aβ1–42 levels exhibited significantly lower serum AHR agonistic activity compared to those with pathological Aβ1–42 levels (*n* = 31). **p < 0.01. **B** Violin/box plot of serum AHR (z-score) in A + T + individuals stratified by dementia status. Age- and sex-matched comparisons revealed significantly lower serum AHR agonistic activity in non-demented (MCI/SCI) individuals (*n* = 44) compared to demented individuals (*n* = 19). No corresponding difference was observed in CSF AHR agonistic activity. *p < 0.05. **C** Scatter plot showing the relationship between serum AHR (z-score) and MMSE performance (z-score) across the entire cohort, stratified by amyloid status (A- in blue, A + in red). Solid lines represent linear regression fits with 95% confidence ribbons. Serum AHR agonistic activity showed a significant but modest inverse correlation with MMSE performance overall (Pearson *r* = −0.20, *p =* 0.019; Spearman ρ = −0.21, *p =* 0.014), with similar trends in both A- (*r* = −0.21, *p =* 0.090) and A + (*r* = −0.25, *p =* 0.038) groups. The interaction between AHR and amyloid status on MMSE was not significant (*p =* 0.562)

Given the observed inverse correlations between CSF Aβ1–42, Aβ1–40, and serum AHR agonistic activity, we proceeded to compare the AHR levels of A + T1 + individuals with normal or elevated Aβ42 and Aβ40 levels (*n* = 32) to those with low Aβ42 levels (*n* = 31; Fig. 3A). In the group with Alzheimer's disease (A + T1 +), serum AHR agonistic activity was significantly lower in individuals with normal CSF Aβ1–42 levels compared to those with pathological Aβ1–42 levels (Δ z-score = −1.89, 95% CI [−3.21, −0.58], *p =* 0.006). There were no differences in age, *p =* 0.918, years of education, *p =* 0.769, or sex, *p =* 0.256, between A + T1 + individuals with normal and pathological Aβ1–42 levels. A + T1 + individual with normal Aβ1–42 levels also showed significantly reduced Serum AHR agonistic activity compared to biomarker negative controls (Δ z-score = −2.45, 95% CI [−3.49, −1.42], *p* < 0.001). In CSF, no significant differences in AHR agonistic activity were observed between A + T1 + individuals with normal Aβ1–42 levels and those with pathological levels (Δ z-score = 0.46 (95% CI [−0.20, 1.12], *p =* 0.169).

### AHR agonistic activity differs between demented and non-demented A + T + individuals in serum but Not CSF

Age- and sex-matched comparisons between non-demented (MCI/SCI, *n* = 44) and demented (*n* = 19) individuals in the A + T + group revealed significantly lower serum AHR agonistic activity in non-demented individuals (Δ z-score = −1.47, 95% CI [−2.62, −0.31]; Fig. 3B). In contrast, no significant difference in CSF AHR agonistic activity was observed between these groups (Δ z-score = 0.15 (95% CI [−0.71, 1.01], *p =* 0.716). In serum, AHR agonistic activity showed a significant but small correlation with z-scored MMSE performance (Pearson *r* = −0.195, *p =* 0.022; Spearman ρ = −0.210, *p =* 0.014; Fig. 3C), whereas no significant association was detected in CSF (Pearson *r* = −0.05, *p =* 0.955; Spearman ρ = −0.05, *p =* 0.955). Given the limited number of demented non-AD cases, subgroup analyses stratified by dementia etiology were not performed.

### AHR agonistic activity was not associated with surrogate markers for systemic or neuroinflammation

**Fig. 4:**
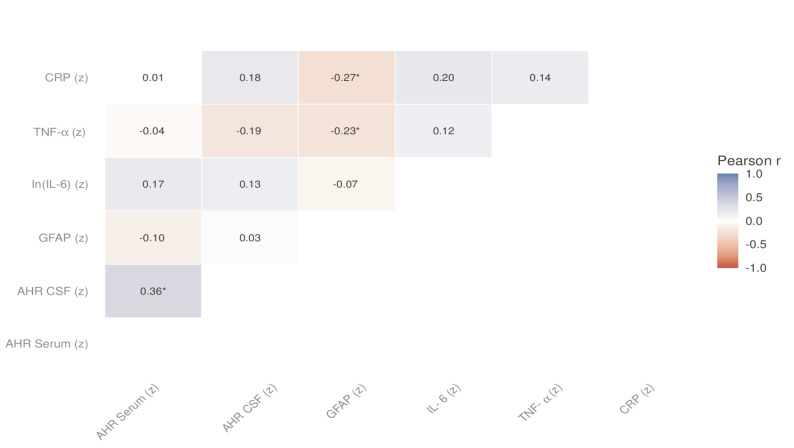
Correlation structure among serum and CSF AHR agonistic activity and inflammation markers Upper-triangle heatmap showing Pearson’s r among z-scored AHR serum, AHR CSF, GFAP, ln(IL-6), TNF-α, and CRP. Cells display r; asterisks denote *p* < 0.05 (two-sided; pairwise complete observations). No robust linear association is observed between AHR (serum or CSF) and systemic or neuroinflammatory markers in this cohort

Neither Pearson nor Spearman correlations showed evidence of linear or rank association with serum or CSF AHR agonistic activity with neuroglial activation marker (GFAP) nor systemic inflammation in a subset of 86 individuals (CRP, IL-6, TNF-α; Fig. 4 and Supplementary Fig. 3). To assess representativeness of this subcohort, baseline characteristics were compared between participants with available inflammation markers (*n* = 86) and those without (*n* = 52). No significant differences were observed in age (*p =* 0.46), education (*p =* 0.54), MMSE (*p =* 0.13), CSF pTau181 (*p =* 0.19), CSF total tau (*p =* 0.37), plasma Aβ1–42 (*p =* 0.08), or Aβ1–42/Aβ1–40 ratio (*p =* 0.64). Plasma Aβ1–40 levels were modestly lower in the inflammation subcohort (−0.69 standard deviations, *p =* 0.012; Supplementary Fig. 5). Using linear regression models, we found no evidence that astrocyte reactivity modulates Aβ-AHR associations. For the primary analysis using median-split GFAP stratification and Aβ42/40 ratio, the Biomarker × GFAP interaction was non-significant (β = 0.100, *p =* 0.561). Similarly, interactions were non-significant for Aβ1–42 (*p =* 0.520), Aβ1–40 (*p =* 0.410), and pTau181 (*p =* 0.844, Supplementary Fig. 6). Results were consistent when using Youden-based GFAP cutoffs and across alternative percentile thresholds (all interaction *p* > 0.10). Effect size analysis revealed no large magnitude effect of combined amyloid and astrocyte status on AHR activity. Continuous GFAP interaction models confirmed these null findings (e.g. Aβ42/40 × GFAP: β = −0.006, *p =* 0.932), indicating results were not driven by dichotomization thresholds. To capture potential non-linear effects of inflammation, we combined CRP, IL-6 and TNF-α into a systemic inflammation first principal component (PC1) and also ran CRP-only sensitivity analyses. GAMs did not support an association between serum AHR and GFAP (smooth p ≈ 0.40), systemic PC1 (p ≈ 0.90), or CRP alone (p ≈ 0.76; Supplementary Table 7). Results were analogous for CSF AHR (systemic PC1 and CRP smooths non-significant; e.g., CRP in CSF model p ≈ 0.58). These null findings were robust to covariate adjustment.

No association was found between CSF or serum AHR agonistic activity and CSF/blood albumin levels, which was employed as a surrogate marker for the permeability of the blood–brain barrier, Serum Pearson *r* = 0.001, *p =* 0.991; Spearman ρ = −0.111, *p =* 0.229 and CSF Pearson *r* = 0.112, *p =* 0.247; Spearman ρ = 0.091, *p =* 0.347.

## Discussion

In this study, we employed a cell-line-based reporter assay to assess serum AHR agonistic activity in patients with mild cognitive impairment (MCI) and dementia, classified according to Alzheimer’s disease (AD) core biomarkers. Our findings reveal a significant reduction in serum AHR agonistic activity between the CSF biomarker negative control group and individuals with pathological Aβ1–42/Aβ1–40 ratio and/or pTau181. The AHR agonistic activity reduction was most pronounced in two distinct groups: (1) individuals with MCI who exhibit elevated pTau levels without positive amyloidopathy biomarkers (the PASSED constellation) [12, 14] and (2) individuals with AD who display normal CSF Aβ1–42 levels when compared to biomarker-negative controls. These reductions occurred independently of detectable neuroinflammation, systemic inflammation, or blood–brain barrier dysfunction. Furthermore, we also found that amyloidopathy modified the pTau181–AHR relationship: In individuals with a non-pathological Aβ1–42/Aβ1–40 ratio (A −), pTau181 was negatively correlated with serum AHR activity. In individuals with a pathological Aβ1–42/Aβ1–40 ratio (A +), this association reversed: pTau181 showed a positive relationship with serum AHR activity, most evident at higher pTau181 levels. In contrast, CSF amyloid peptides (Aβ1–40, Aβ1–42) showed consistent negative correlations with serum AHR activity regardless of amyloid aggregation status.

In contrast to previous investigations focusing on individual AHR agonists or antagonists—such as indole derivatives [21, 22]—we examined the overall AHR agonistic system, which reflects the net activity generated by both agonistic and antagonistic compounds from diverse sources.

Interestingly, despite the robust differences observed in serum AHR activity, no significant differences were detected in the CSF. This discrepancy may be attributed to differential compartmentalization of AHR ligands, where peripheral alterations are not directly mirrored in the central nervous system. Additionally, technical limitations in measuring AHR activity in the CSF and potential compensatory regulatory mechanisms within the CNS might obscure group differences.

Our data also underscore the complexity of biomarker changes in AD. In non-demented individuals, isolated elevated pTau181 levels without a concomitant reduction in the Aβ ratio (A-T1 +, PASSED), together with alterations in Aβ1–42 and Aβ1–40, do not necessarily correlate with significant cognitive decline [12, 14, 23]. This observation raises questions about the pathological significance of these alterations in the context of AD. Notably, reduced serum AHR agonistic activity was observed not only in PASSED individuals but also in AD patients with normal CSF Aβ1–42 levels. Given that AD patients with normal Aβ1–42 levels have been associated with longer survival compared to those with pathological Aβ1–42 levels, one might hypothesize that reduced serum AHR activity could be linked to a slower progression of cognitive decline [24, 25]. However, since lower AHR activity has been reported to be associated with increased neuroinflammation [5] —typically correlated with faster decline—GFAP and IL-6, TNF-alpha and CRP blood levels as surrogate markers for neuroinflammation and systemic inflammation showed no significant association with AHR serum and CSF levels in this cohort. The absence of correlation between AHR agonistic activity and inflammation markers (GFAP, CRP, IL-6, TNF-α) in a subcohort challenges the interpretation that reduced AHR activity reflects active neuroinflammation. This finding may suggest that: (a) AHR alterations precede detectable inflammatory changes in early-stage disease, (b) AHR activity relates to amyloid metabolism through inflammation-independent pathways, or (c) our cross-sectional design failed to capture dynamic relationships that emerge over time. The correlation with Aβ1–42, pTau181 and Aβ1–40 but not with inflammation markers suggests AHR may be more closely linked to amyloid production or clearance mechanisms than to inflammatory cascades. Consistent with this, our findings provide human correlative evidence that complements preclinical mechanistic studies. Qian et al. demonstrated that AhR activation upregulates neprilysin (NEP), a major Aβ-degrading enzyme, through direct transcriptional regulation [10]. Additionally, AHR agonistic activity might modulate γ-secretase function by influencing membrane lipid composition or alter autophagic clearance, thereby affecting soluble amyloid-beta levels [10, 26–28].

Assuming that the stages in which measured reduced AHR agonist activity are entities with a good prognosis or early biological stages of Alzheimer's disease, this opens up perspectives on when modulation of AHR is particularly useful [29]. AHR agonists derive from diverse origins, including environmental toxins, dietary elements, and endogenous metabolic processes. For instance, cruciferous vegetables supply AHR ligands—such as 3,3’-diindolylmethane and indole-3-carbinol—with documented anti-inflammatory properties, while tryptophan metabolism and microbiome-associated enzymatic pathways also yield AHR ligands [9, 30]. Alterations in the gut microbiome, endogenous metabolic shifts, and differences in excretion pathways may all contribute to this phenomenon [9]. In addition, AHR regulates both peripheral and central immune responses by modulating the function of dendritic cells, T cells, astrocytes, and microglia, which are known to be altered in AD. Thus, AHR might link the effects of dietary and environmental factors to disease progression by influencing protein degradation [9, 31]. Recent evidence also suggests that adherence to a Mediterranean diet—which can influence AHR signaling—confers protection against AD, highlighting the significant impact of dietary and environmental factors on disease progression [31–33].

### Limitations

Notwithstanding these observations, our study has several limitations. First, pre-analytical factors such as sample processing, storage conditions, and potential degradation of AHR ligands may have influenced the results, although these factors likely affected both patient and control samples similarly. Second, the use of a transient rather than a stable transfection system may introduce variability in the measurement of AHR activity, despite prior evidence suggesting comparable net activity between the two approaches [34]. Third, the lack of an independent replication cohort and the cross-sectional design of the study limit the generalizability and temporal interpretation of our findings. Fourth, the relatively small subgroup sizes (particularly A + T1 −, *n* = 9) limit statistical power and increase the risk of both false-positive and false-negative findings. Fifth, while we measured multiple inflammation markers, the lack of association with AHR suggests either that our hypothesis linking AHR to neuroinflammation requires refinement, or that inflammation-AHR relationships are temporally dynamic and not captured by cross-sectional measurement in the subcohort of 86 individuals. Future studies should include independent validation cohorts and longitudinal data to confirm our results and better define the dynamic role of AHR signaling in Alzheimer’s disease progression.

## Conclusion

In conclusion, our study demonstrates that serum AHR agonistic activity is significantly lower in non-demented subjects with isolated tau pathology (A-T1 +) and in AD patients (A + T1 +) with preserved Aβ1–42 levels despite pathological Aβ1–42/Aβ1–40 ratios. These alterations correlate with CSF amyloid peptides Aβ1–42 and Aβ1–40 but show amyloidopathy-dependent modulation of pTau181-AHR relationships. Future studies should employ larger, longitudinal cohorts to validate these results and elucidate the underlying mechanisms, while exploring non-toxic AHR agonists—such as synthetic small molecules or probiotics—as promising treatment options.

## Supplementary Information

Supplementary Material 1.

Supplementary Material 2: Supplementary Figure 1. Correlation analysis between serum and CSF AHR agonistic activity and CSF biomarkers Aβ1–42, pTau181, and total Tau Upper-triangle heatmap showing Pearson’s r (A) and Spearman´s r among z-scored AHR serum, AHR CSF, age, education, eGFR, creatinine, GOT, GPT, γGT, HbA1c, and zMMSE (pairwise complete observations). *p < 0.05.

Supplementary Material 3: Supplementary Figure 2. Correlation structure among serum and CSF AHR agonistic activity and inflammation markers (Spearman) Upper-triangle heatmap showing Spearman’s ρ for the same panel as Figure 2 among z-scored AHR serum, AHR CSF, GFAP, ln(IL-6), TNF-α, and CRP. Cells display r; *p < 0.05 (two-sided; pairwise complete observations).

Supplementary Material 4: Supplementary Figure 3. Baseline characteristics comparison between participants with and without available inflammation marker data Violin/box plots comparing key demographic, cognitive, and biomarker characteristics between participants with available inflammation markers (A, *n =* 86) and those without available data (N/A, *n=* 52). Jittered points represent individual participants; box plots display median and interquartile range. CSF Aβ1-40 z-scores were modestly but significantly lower in the inflammation subcohort (*p* = 0.01, d = -0.69). P-values derived from two-sided t-tests; Cohen's d represents standardized effect sizes. This analysis demonstrates that the inflammation subcohort is broadly representative of the full study sample, with only minor differences in CSF Aβ1-40 levels. *p < 0.05.

## Data Availability

Data that support the study’s findings are available from the corresponding author upon reasonable request and after approval of the data coordinating center.

## Abbreviations

Aβ: Amyloid beta
AD: Alzheimer’s Disease
AHR: Aryl Hydrocarbon Receptor
AT: Amyloid/Tau classification
ATN: Amyloid/Tau/Neurodegeneration classification
ANCOVA: Analysis of Covariance
ANOVA: Analysis of Variance
BBB: Blood-brain barrier
CI: Confidence Interval
CNS: Central Nervous System
CSF: Cerebrospinal Fluid
DMEM: Dulbecco’s Modified Eagle Medium
MCI: Mild Cognitive Impairment
MMSE: Mini-Mental State Examination
MRI: Magnetic Resonance Imaging
NDD: Neurodegenerative Disease
PASSED: PTau-levels and Aβ Surge with Subtle Cognitive Deterioration
pTau181: Phosphorylated tau at threonine 181
SCI: Subjective Cognitive Impairment
SD: Standard Deviation
SNAP: Suspected Non-Alzheimer’s Disease Pathophysiology
SPSS: Statistical Package for the Social Sciences
