# Supplementary material for: Reduced serum AHR agonistic activity reflects amyloid dysregulation in AT1 subtypes of Alzheimer’s disease
Source: Alzheimers Res Ther. 2026 Feb 6;18:47. doi: 10.1186/s13195-026-01978-w (PMC12930576; doi:10.1186/s13195-026-01978-w)
Supplement: Supplementary file 1 — Supplementary Material 1. [file 13195_2026_1978_MOESM1_ESM.docx]

**Supplementary Tables 1-7:**

| **Supplementary Table 1:** | | |  |  |  |  |  |  |  |  |  |  |  |  |  |  | |  |
| --- | --- | --- | --- | --- | --- | --- | --- | --- | --- | --- | --- | --- | --- | --- | --- | --- | --- | --- |
| **Characteristics of individuals with subjective cognitive impairment (SCI), mild cognitive impairment (MCI) and dementia.** | | | | | | | | | | | |  |  |  |  |  | |  |
|  |  |  |  |  |  |  |  |  |  |  |  |  |  |  |  |  | |  |
|  |  | **A-T-** | | | | **A+T+** | | | | **A-T+** | | | | **A+T-** | | | | **p-value** |
|  |  | **n** | | **%** | | **n** | | **%** | | **n** | | **%** | | **n** | | **%** | |  |
| **n [female]** | **SCI** | 6 | [3] | 26 | [13] | 2 | [0] | 9 | [0] | 9 | [3] | 39 | [13] |  |  |  |  | N/A |
|  | **MCI** | 29 | [14] | 20 | [10] | 42 | [21] | 29 | [14] | 16 | [9] | 11 | [6] | 9 | [5] | 6 | [3] | 0.950 |
|  | **Dementia** | 4 | [1] | 11 | [3] | 19 | [11] | 51 | [30] | 2 | [0] | 5 | [0] |  |  |  |  | N/A |
|  |  |  |  |  |  |  |  |  |  |  |  |  |  |  |  |  |  |  |
|  |  | **mean** | | **SD** | | **mean** | | **SD** | | **mean** | | **SD** | | **mean** | | **SD** | |  |
| **Age at Visit [years]** | **SCI** | 64 | | 8 | | 73 | | 13 | | 64 | | 7 | |  | |  | | N/A |
|  | **MCI** | 65 | | 8 | | 70 | | 10 | | 65 | | 8 | | 74 | | 9 | | 0.154 |
|  | **Dementia** | 76 | | 11 | | 69 | | 9 | | 74 | | 11 | |  | |  | | N/A |
| **Education [years]** | **SCI** | 13 | | 3 | | 18 | | 3 | | 14 | | 3 | |  | |  | | N/A |
|  | **MCI** | 14 | | 3 | | 14 | | 3 | | 13 | | 3 | | 13 | | 5 | | 0.817 |
|  | **Dementia** | 11 | | 2 | | 12 | | 1 | | 12 | | 1 | |  | |  | | N/A |
| **MMSE** | **SCI** | 29 | | 1 | | 29 | | 0 | | 29 | | 1 | |  | |  | | N/A |
|  | **MCI** | 27 | | 2 | | 26 | | 2 | | 27 | | 2 | | 26 | | 2 | | 0.182 |
|  | **Dementia** | 18 | | 7 | | 21 | | 4 | | 23 | | 2 | |  | |  | | N/A |
| **Aβ 1-42 [pg/ml]** | **SCI** | 992 | | 192 | | 655 | | 40 | | 1589 | | 466 | |  | |  | | N/A |
|  | **MCI** | 1138 | | 363 | | 643 | | 202 | | 1361 | | 293 | | 449 | | 166 | | **< 0.001** |
|  | **Dementia** | 990 | | 240 | | 551 | | 275 | | 743 | | 230 | |  | |  | | N/A |
| **Aβ 1-40 [pg/ml]** | **SCI** | 10661 | | 3401 | | 14355 | | 617 | | 18251 | | 4017 | |  | |  | | N/A |
|  | **MCI** | 11511 | | 3083 | | 16890 | | 6206 | | 16766 | | 3063 | | 8099 | | 3512 | | **< 0.001** |
|  | **Dementia** | 10044 | | 1294 | | 12834 | | 4817 | | 9450 | | 2263 | |  | |  | | N/A |
| **Aβ1-42/Aβ1-40** | **SCI** | 0.10 | | 0.02 | | 0.05 | | < 0.01 | | 0.08 | | 0.01 | |  | |  | | N/A |
|  | **MCI** | 0.10 | | 0.01 | | 0.04 | | 0.01 | | 0.08 | | 0.02 | | 0.04 | | 0.01 | | **< 0.001** |
|  | **Dementia** | 0.10 | | 0.01 | | 0.04 | | < 0.01 | | 0.08 | | < 0.01 | |  | |  | | N/A |
| **pTau181 [pg/ml]** | **SCI** | 35 | | 14 | | 80 | | 7 | | 73 | | 8 | |  | |  | | N/A |
|  | **MCI** | 36 | | 8 | | 110 | | 50 | | 66 | | 15 | | 34 | | 14 | | **< 0.001** |
|  | **Dementia** | 37 | | 5 | | 135 | | 57 | | 56 | | 1 | |  | |  | | N/A |
| **total tau [pg/ml]** | **SCI** | 211 | | 65 | | 376 | | 68 | | 376 | | 67 | |  | |  | | N/A |
|  | **MCI** | 258 | | 77 | | 486 | | 417 | | 486 | | 417 | | 223 | | 76 | | **< 0.001** |
|  | **Dementia** | 303 | | 71 | | 645 | | 378 | | 645 | | 378 | |  | |  | | N/A |

**Supplementary Table 2:**

**Characteristics of A+T+ indviduals with normal versus pathological Aβ 1-42 levels .**

|  | **Normal Aβ 1-42** | | | | **Pathological Aβ 1-42** | | | | **p-value** |
| --- | --- | --- | --- | --- | --- | --- | --- | --- | --- |
|  | **n** | | **%** | | **n** | | **%** | |  |
| **n [female]** | 32 | [14] | 28.6 | [22.2] | 31 | [18] | 20.6 | [28.6] | 0.255 |
|  |  |  |  |  |  |  |  |  |  |
|  | **mean** | | **SD** | | **mean** | | **SD** | |  |
| **Age at Visit [years]** | 70 | | 7 | | 70 | | 11 | | 0.864 |
| **Education [years]** | 13 | | 3 | | 14 | | 3 | | 0.895 |
| **MMSE** | 26 | | 2 | | 24 | | 5 | | 0.009 |
| **Aβ 1-42 [pg/ml]** | **775** | | **395** | | **451** | | **84** | | **< 0.001** |
| **Aβ 1-40 [pg/ml]** | **18954** | | **5353** | | **12111** | | **4436** | | **< 0.001** |
| **pTau181 [pg/ml]** | 114 | | 53 | | 119 | | 53 | | 0.709 |
| **total tau [pg/ml]** | 735 | | 395 | | 806 | | 432 | | 0.496 |

**Supplementary Table 3:**

**Results of ANCOVAs: unadjusted and adjusted serum AHR activity levels in non-demented subjects by ATN classification**

|  | **M** | | **SE** | | **95% confidence intervall** | | | |
| --- | --- | --- | --- | --- | --- | --- | --- | --- |
|  |  | |  | | **Lower Bound** | | **Upper bound** | |
|  | **Adj.** | **Unadj.** | **Adj.** | **Unadj.** | **Adj.** | **Unadj.** | **Adj.** | **Unadj.** |
| **A-T1-** | 0.28a | 0.22 | 0.39 | 0.38 | -0.49 | -0.55 | 1.05 | 0.97 |
| **A+T1+** | **-1.81a** | **-1.70** | **0.35** | **0.34** | **-2.50** | **-2.38** | **-1.11** | **-1.02** |
| **A-T1+** | **-2.33a** | **-2.41** | **0.47** | **0.45** | **-3.31** | **-3.31** | **-1.40** | **-1.51** |
| **A+T1-** | -1.52a | -1.40 | 0.77 | 0.76 | -2.90 | -2.90 | 0.01 | 0.10 |

a. The covariates in the model are calculated using the following values: Age at Visit [y] = 67.6, Education [y] = 13.8, zMMSE = -1.65.

**Supplementary Table 4:**

**Summary of linear regression models for serum AHR activity vs CSF amyloid and pTau markers, by Amyloidopathy (A−/A+)**

| **Biomarker** | **A- β (SE)** | **A- p-value** | **A+ β (SE)** | **A+ p-value** |  | **Interaction β** | **Interaction p** |
| --- | --- | --- | --- | --- | --- | --- | --- |
| **pTau181** | **-0.54 (0.12)** | **<0.001** | **+0.11 (0.05)** | **0.025** |  | **0.65** | **<0.0001** |
| **Aβ1–42** | **-0.55 (0.19)** | **0.006** | **-1.24 (0.37)** | **0.002** |  | **0.69** | **0.10** |
| **Aβ1–40** | **-0.66 (0.24)** | **0.008** | **-0.34 (0.22)** | **0.13** |  | **-0.32** | **0.32** |
| Aβ42/40 | -0.08 (0.23) | 0.73 | -0.29 (0.28) | 0.31 |  | 0.21 | 0.56 |

1. All models adjusted for age, sex, education, MMSE, HbA1c, creatinine, and GPT.

**Supplementary Table 5:**

**Summary of adjusted GAMs for serum AHR agonistic activity vs CSF pTau markers, by Amyloidopathy (A−/A+)**

| **Model (smooth)** | **n** | **edf** | **F** | **p** | **Adj. R²** | **Dev. explained** | **AIC** |
| --- | --- | --- | --- | --- | --- | --- | --- |
| **A- group** | **66** | **1.77** | **12.84** | **<0.001** | **0.457** | **53.2%** | **242.6** |
| **A+ group** | **71** | **1.35** | **2.70** | **0.004** | **0.207** | **29.0%** | **295.4** |
| Pooled (main effects) | 137 | 2.38 | 12.10 | <0.001 | 0.264 | 32.1% | 594.0 |
| Interaction (A- smooth) | 137 | 1.03 | 4.17 | <0.001 | 0.221 | 28.0% | 602.3 |
| Interaction (A+ smooth) | 137 | 1.59 | 5.47 | <0.001 |  |  |  |
| Overlap: A- | 66 | 1.69 | 8.61 | <0.001 | 0.359 |  | 415.4 |
| Overlap: A+ | 37 | 0.00 | 0.00 | 0.872 |  |  |  |

a. all models use thin-plate splines (k=4), REML, covariates: age (smooth), sex, education, eGFR, GPT, HbA1c, MMSE.

**Supplementary Table 6:**

**Summary of linear regression models for serum and CSF AHR activity vs CSF amyloid and pTau markers, by GFAP statuts (GFAP−/GFAP+)**

| **Outcome** | **CSF Biomarker** | **GFAP cutoff** | **R2** | **Interaction β** | **Interaction p** | **GFAP- β** | **GFAP- p** | **GFAP+ β** | **GFAP+ p** |
| --- | --- | --- | --- | --- | --- | --- | --- | --- | --- |
| Serum AHR | Aβ1-40 | Mdn | 0.155 | -0.236 | 0.410 | -0.194 | 0.382 | -0.429 | 0.236 |
|  |  | YI | 0.139 | 0.135 | 0.629 | -0.408 | 0.046 | -0.273 | 0.428 |
|  | Aβ1-42 | Mdn | 0.205 | -0.142 | 0.520 | -0.331 | 0.037 | -0.473 | 0.083 |
|  |  | YI | 0.175 | 0.174 | 0.543 | -0.432 | 0.003 | -0.258 | 0.420 |
|  | Aβ42/Aβ40 | Mdn | 0.117 | 0.100 | 0.561 | -0.191 | 0.169 | -0.091 | 0.681 |
|  |  | YI | 0.113 | 0.317 | 0.118 | -0.234 | 0.074 | 0.083 | 0.728 |
|  | pTau | Mdn | 0.094 | -0.014 | 0.844 | 0.000 | 0.998 | -0.014 | 0.884 |
|  |  | YI | 0.090 | 0.001 | 0.985 | -0.028 | 0.659 | -0.026 | 0.778 |
| CSF AHR | Aβ1-40 | Mdn | 0.131 | -0.272 | 0.251 | 0.347 | 0.066 | 0.075 | 0.804 |
|  |  | YI | 0.103 | -0.074 | 0.751 | 0.209 | 0.224 | 0.135 | 0.639 |
|  | Aβ1-42 | Mdn | 0.105 | -0.101 | 0.604 | 0.165 | 0.254 | 0.064 | 0.792 |
|  |  | YI | 0.091 | 0.190 | 0.462 | 0.048 | 0.704 | 0.238 | 0.409 |
|  | Aβ42/Aβ40 | Mdn | 0.088 | 0.169 | 0.242 | -0.099 | 0.383 | 0.070 | 0.701 |
|  |  | YI | 0.081 | 0.203 | 0.285 | -0.111 | 0.292 | 0.092 | 0.671 |
|  | pTau | Mdn | 0.090 | -0.020 | 0.725 | 0.011 | 0.831 | -0.009 | 0.909 |
|  |  | YI | 0.078 | -0.018 | 0.756 | 0.012 | 0.814 | -0.006 | 0.941 |

1. All models adjusted for age, sex, education, MMSE b. Mdn = median c. YI = Youden Index

**Supplementary Table 7:**

**Summary of adjusted GAMs for serum AHR agonistic activity vs neuroinflammation and systemic inflammation markers**

| **Predictor (smooth term)** | **edf** | **F** | **p-value** | **adj. R²** | **Deviance explained** | **AIC*** |
| --- | --- | --- | --- | --- | --- | --- |
| **GFAP** | 0.00 | 0.00 | 0.396 | 0.038 | 0.119 | 252.0 |
| **Systemic PC1 (CRP, IL-6, TNFα)** | 0.00 | 0.00 | 0.898 | 0.030 | 0.114 | 243.0 |
| **CRP (alone)** | 0.00 | 0.00 | 0.758 | 0.038 | 0.119 |  |
|  |  |  |  |  |  |  |
