# Supplementary figures and images for: Reduced serum AHR agonistic activity reflects amyloid dysregulation in AT1 subtypes of Alzheimer’s disease
Source: Alzheimers Res Ther. 2026 Feb 6;18:47. doi: 10.1186/s13195-026-01978-w (PMC12930576; doi:10.1186/s13195-026-01978-w)

A

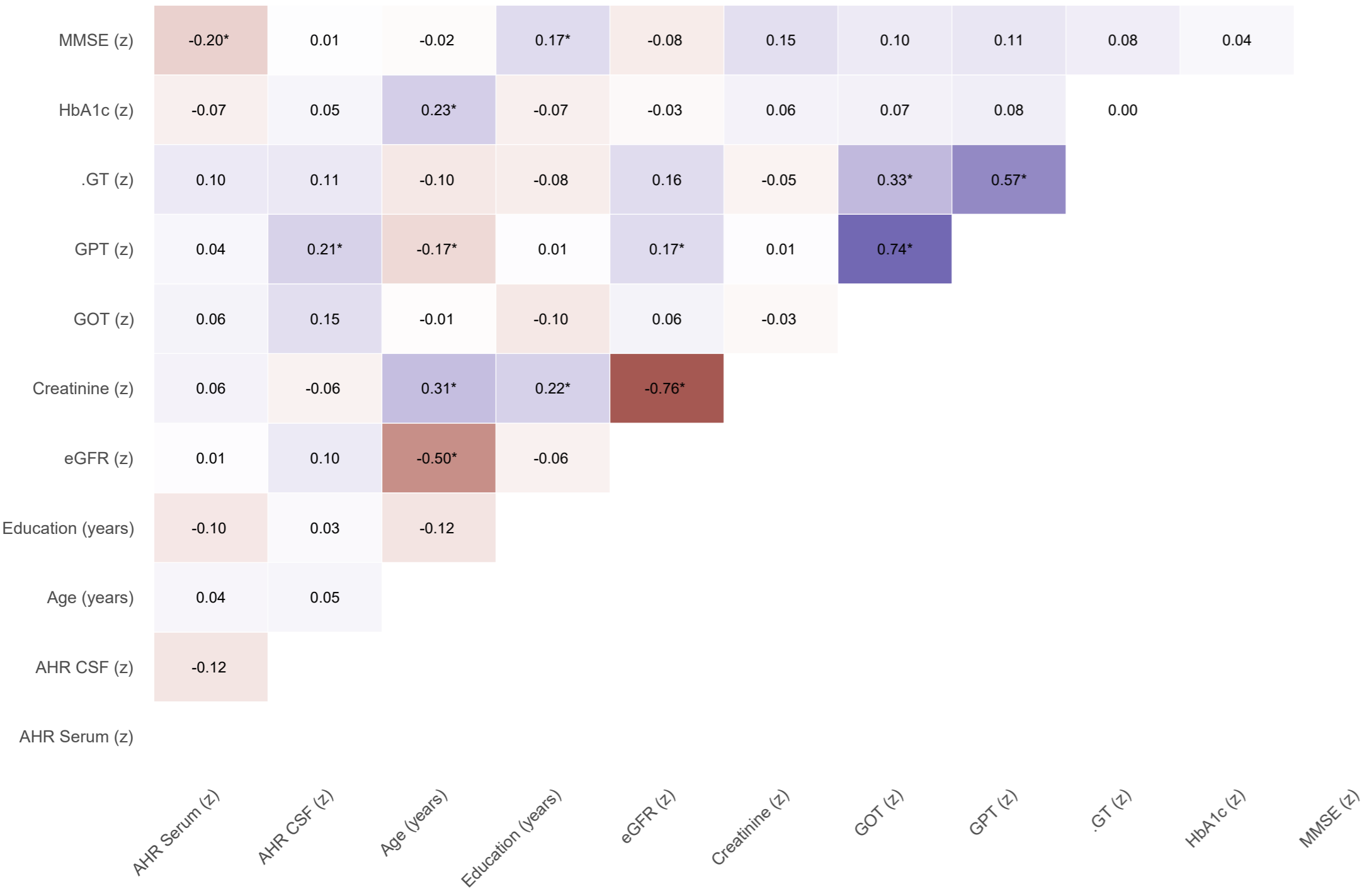

B

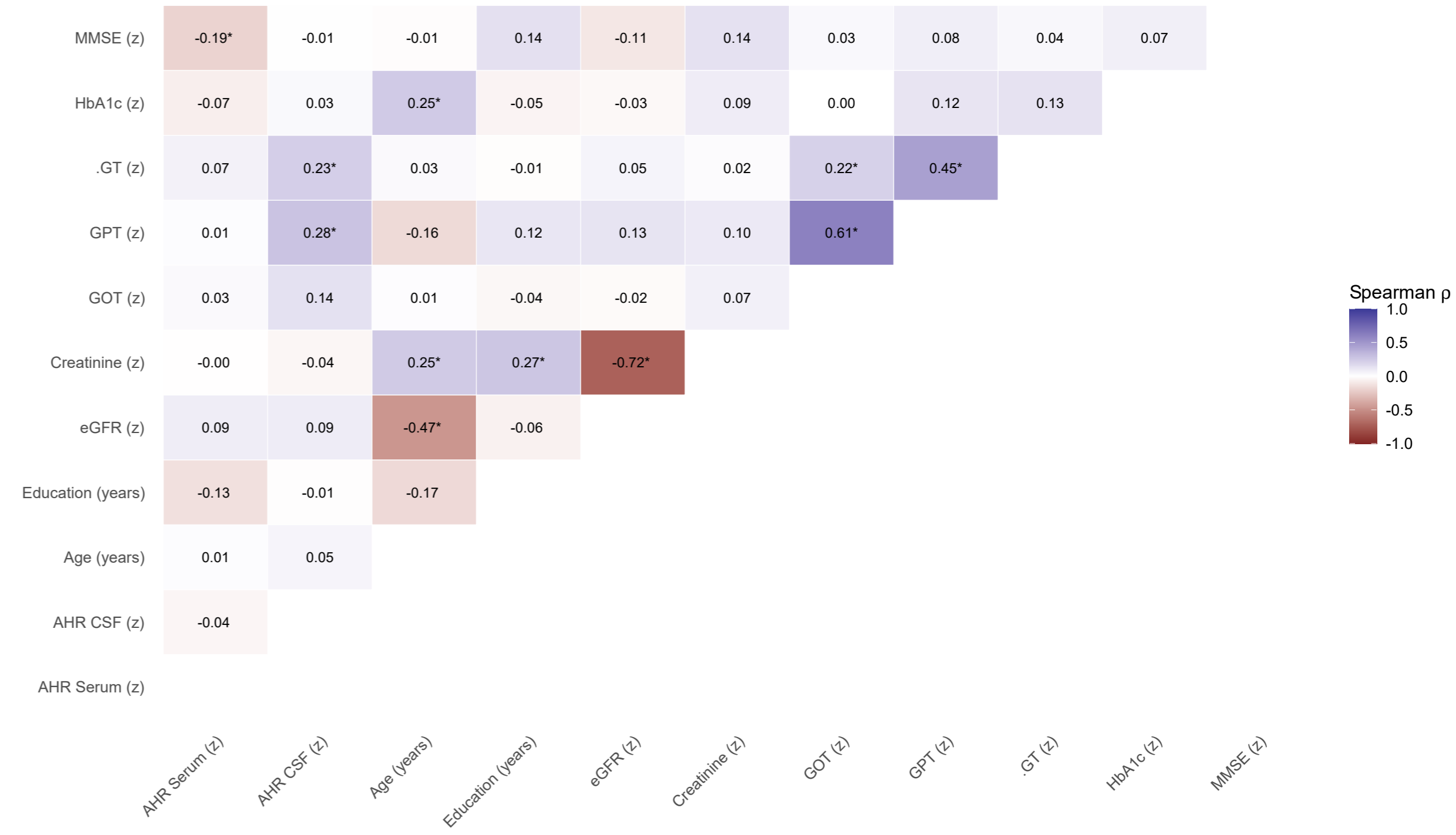

Supplement: Supplementary file 2 — Supplementary Material 2: Supplementary Figure 1. Correlation analysis between serum and CSF AHR agonistic activity and CSF biomarkers Aβ1–42, pTau181, and total Tau Upper-triangle heatmap showing Pearson’s r (A) and Spearman´s r among z-scored AHR serum, AHR CSF, age, education, eGFR, creatinine, GOT, GPT, γGT, HbA1c, and zMMSE (pairwise complete observations). *p < 0.05. [file 13195_2026_1978_MOESM2_ESM.pdf]

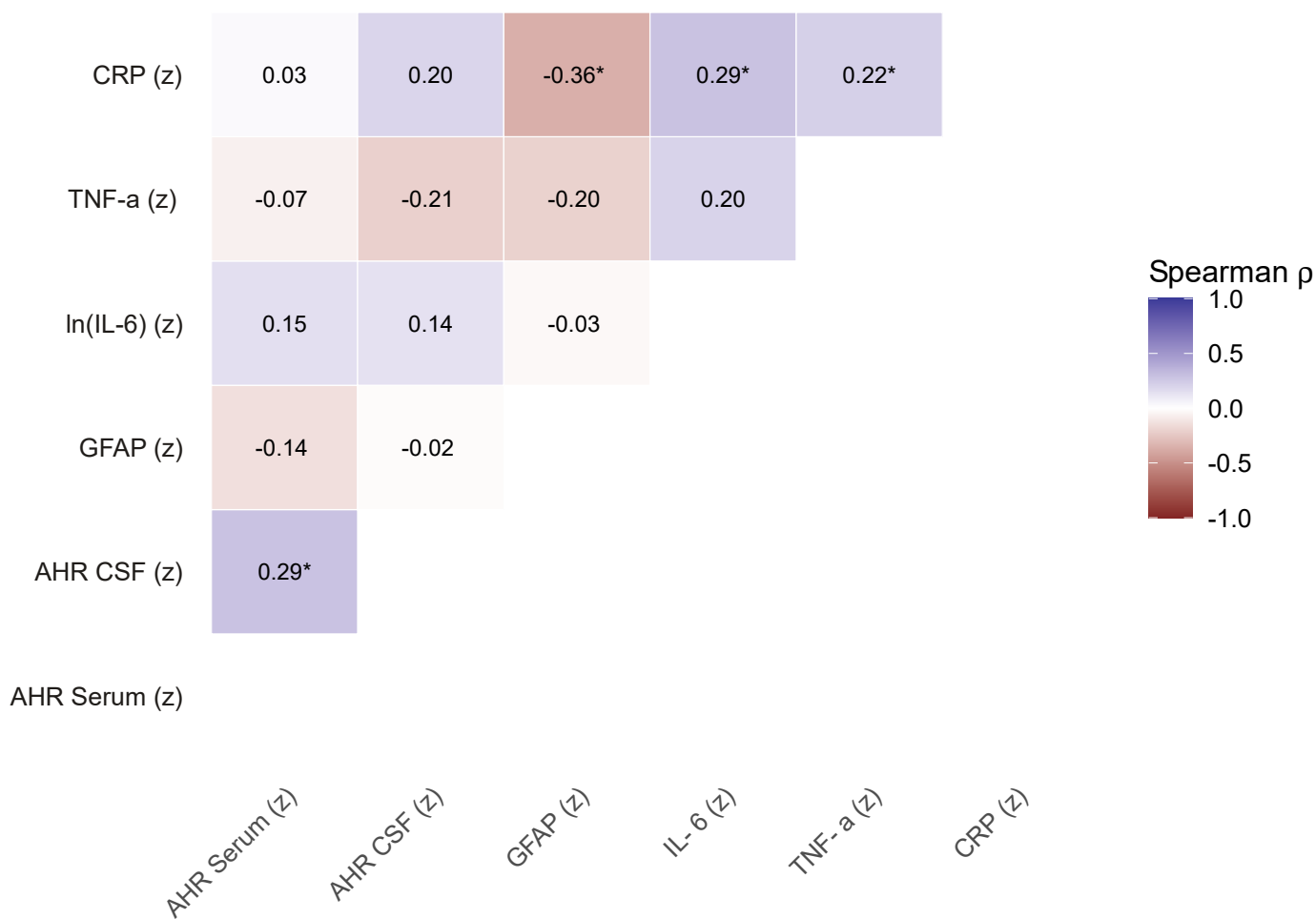

Supplement: Supplementary file 3 — Supplementary Material 3: Supplementary Figure 2. Correlation structure among serum and CSF AHR agonistic activity and inflammation markers (Spearman) Upper-triangle heatmap showing Spearman’s ρ for the same panel as Figure 2 among z-scored AHR serum, AHR CSF, GFAP, ln(IL-6), TNF-α, and CRP. Cells display r; *p < 0.05 (two-sided; pairwise complete observations). [file 13195_2026_1978_MOESM3_ESM.pdf]
