# Supplementary material for: Reduced serum AHR agonistic activity reflects amyloid dysregulation in AT1 subtypes of Alzheimer’s disease
Source: Alzheimers Res Ther. 2026 Feb 6;18:47. doi: 10.1186/s13195-026-01978-w (PMC12930576; doi:10.1186/s13195-026-01978-w)
Supplement: Supplementary file 4 — Supplementary Material 4: Supplementary Figure 3. Baseline characteristics comparison between participants with and without available inflammation marker data Violin/box plots comparing key demographic, cognitive, and biomarker characteristics between participants with available inflammation markers (A, n = 86) and those without available data (N/A, n= 52). Jittered points represent individual participants; box plots display median and interquartile range. CSF Aβ1-40 z-scores were modestly but significantly lower in the inflammation subcohort (p = 0.01, d = -0.69). P-values derived from two-sided t-tests; Cohen's d represents standardized effect sizes. This analysis demonstrates that the inflammation subcohort is broadly representative of the full study sample, with only minor differences in CSF Aβ1-40 levels. *p < 0.05. [file 13195_2026_1978_MOESM4_ESM.pdf]

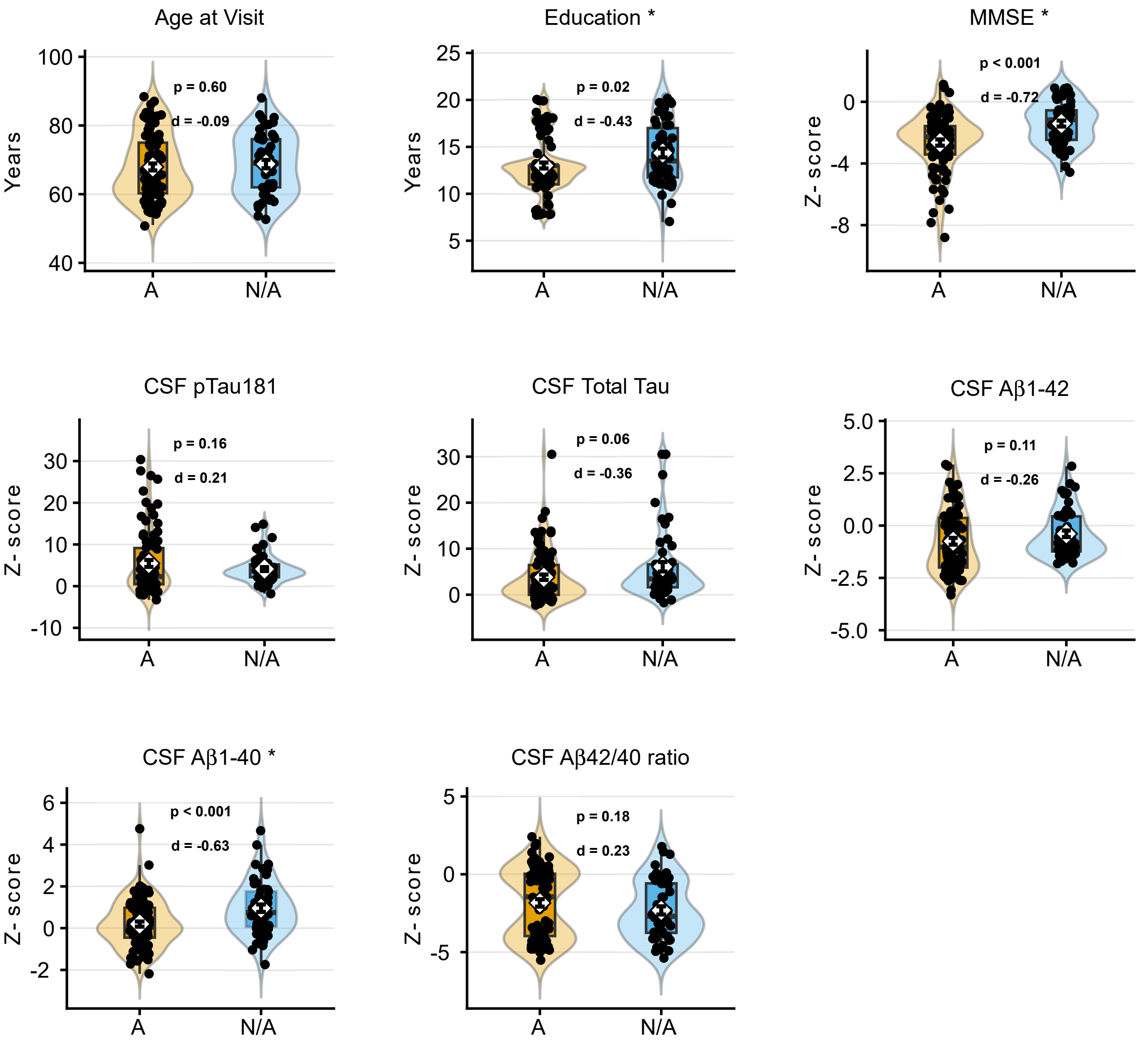

A = Available (n=86); N/A = Not available (n=52)
